# Effect of 8-week n-3 fatty-acid supplementation on oxidative stress and inflammation in middle- and long-distance running athletes: a pilot study

**DOI:** 10.1186/s12970-020-00391-4

**Published:** 2020-11-11

**Authors:** Daniela Buonocore, Manuela Verri, Andrea Giolitto, Enrico Doria, Michele Ghitti, Maurizia Dossena

**Affiliations:** 1 Department of Biology and Biotechnology “Lazzaro Spallanzani”, University of Pavia, Via Ferrata, 9-27100 Pavia (PV), Italy; 2 Department of Earth and Environmental Sciences (DSTA) – Unit of Statistical Analyses (UNISTAT), University of Pavia, Via Ferrata, 9-27100 Pavia (PV), Italy

**Keywords:** Docosahexaenoic acid, Eicosapentaenoic acid, Inflammation, Long-chain n-3 polyunsaturated fatty acids, Nutraceuticals, Oxidative status, Physical exercise

## Abstract

**Background:**

Long-chain n-3 polyunsaturated fatty acids, such as eicosapentaenoic acid (EPA) and docosahexaenoic acid (DHA), may alter oxidative status and immune function after exercise. The aim of this pilot study was to determine the probable association between n-3 supplementation and physical exercise, observing the variations in markers of oxidative stress and inflammation.

**Methods:**

Thirty-nine subjects of both sexes aged 17–30 years were divided into two groups: 1) (*n* = 21) trained Athletes; 2) (*n* = 18) Sedentary subjects. All subjects were given about 4 g/day of n-3 supplementation, rich in EPA and DHA, for 8 weeks. Blood, saliva and urine samples were collected pre- (T0) and post- (T1) supplementation. Hematological parameters (tryglicerides, total cholesterol, HDL, CPK, LDH, HGH, IGF-1), oxidative markers (MDA, 8-OHdG, PCc), antioxidant parameters (GPx, SOD, CAT, DPPH scavenger), exercise-induced stress markers (testosterone and cortisol) and an inflammatory marker (TNF-α) were measured. All tests were two-sided and a *p*-value of less than 0.05 was considered as statistically significant.

**Results:**

The **results** showed that MDA and TNF-αmean values significantly decreased after supplementation in both Athletes and Sedentary subjects: variation was greater in Athletes than in Sedentary control subjects. Generally, our results suggested that supplementation with n-3 PUFAs created a synergic variation in the parameters from a baseline state (T0) to a treated state after supplementation (T1), in terms of size and modality, which was significantly different in Athletes compared to Sedentary subjects.

**Conclusion:**

In **conclusion**, supplementation with about 4 g/day of n-3 PUFAs, rich in EPA and DHA, for 8 weeks, seemed to be effective in counteracting some parameters involved in oxidative stress and inflammation, induced by acute strenuous physical exercise.

## Background

Physical training and training sessions for middle- and long-distance running, performed by agonistic/sports professional athletes, are examples of intense and prolonged physical exertion. During the period of physical preparation and training sessions, as well as during competition, two fundamental qualities are important in order for an athlete to stand out: speed and endurance, which are closely related to endurance sport performance and to the maximal lactate steady state (MLSS) [1]. Referring to performance, it is well recognized that exhaustive exercise can lead to muscle fatigue, delayed-onset muscle soreness, and a decrement in performance; particularly, the acute strenuous prolonged exercise that is performed during middle- and long-distance running is combined with an increase in reactive oxygen species (ROS) production (including free radicals), changes in blood antioxidant status, and an increase in inflammatory responses. All these events may result in oxidative stress or generally cause DNA damage, lipid peroxidation with increased levels of toxic aldehydes, fatigue, reduction of heme-iron blood levels and release of pro-inflammatory cytokines (interleukins) [2–5].

Therefore, oxidative stress and inflammation induced by acute strenuous physical exercise should be limited in order for athletes to maintain a state of well-being and health, as they are constantly exposed to stress conditions and to the risk of overreaching, which is considered as an accumulation of training load, leading to performance decrements that require days to weeks for recovery. Moreover, systemic inflammation and subsequent effects on the central nervous system may cause a worsening of this syndrome, known as overtraining syndrome (OTS), including depressed mood, central fatigue, and resultant neuro-hormonal changes [6].

The use of food supplements has been considered as a valid method of reducing oxidative stress and systemic inflammation [7]. Omega-3 or n-3 polyunsatured fatty acids (PUFAs), such as the eicosapentaenoic acid (EPA) and docosahexaenoic acid (DHA), are nutrients that seem to possess both antioxidant and anti-inflammatory effects, primarily through their effects on the neutrophil and macrophage components [8] and on mediators of acute inflammation, such as D-series resolvins, protectins and maresins [9, 10]. EPA and DHA can cause dual inhibition of cyclo-oxygenase and lipoxygenase pathways for metabolism of arachidonic acid (AA) [11]. Regarding the molecular mechanism, it is known that n-3 PUFAs affect the inflammatory cytochine gene expression, modifying the activity of the transcription nuclear factor – ҡB and the peroxisome proliferator-activated receptor (PPAR-γ) [12].

It has been suggested that the ingestion of 1–2 g/day of EPA and DHA, at a ratio of 2:1 EPA to DHA, may be beneficial for athletes’ health by neutralizing exercise-induced inflammation [13]. However, the human data are inconclusive as to whether n-3 PUFA supplementation at this dosage is effective in attenuating the inflammatory and immunomodulatory response to exercise [14, 15]. Some authors do not find merit in such a recommendation, concluding that n-3 PUFA supplementation might be a potential aid, i.e. athletes with exercise-induced bronchoconstriction [16], and other groups may benefit (i.e. strength athletes), but there is insufficient data from high quality studies in this area [17]. Therefore, the main aim of the present longitudinal, prospective, pilot study was to determine the association between long-chain n-3 PUFA supplementation, rich in EPA and DHA, and physical exercise, observing the probable variations of markers of lipid profile, muscle workload, oxidative and antioxidant status, steroid hormones as markers of exercise-induced stress, and inflammatory status in middle- and long-distance running Athletes. It has been hypothesized that EPA/DHA would attenuate the exercise-induced rise in biomarkers, compared to a control group of sedentary subjects. As far as we know, this study is the first of its kind in this sport specialization. The second aim was to study whether a synergistic effect exists for the interaction between two stimuli, physical exercise and n-3 supplementation, on the aforementioned variables.

## Materials and methods

### Subjects

Thirty-nine healthy subjects of both sexes aged 17–30 years (average 23.80 ± 5.88) volunteered to participate in this study. All of the subjects resided in Italy and all but two of them (from north Africa) were Italian with a Mediterranean diet. They were divided into the following two groups:

1) (*n* = 21) Trained Athletes, middle-distance runners (800 m, 1500 m, 3000 m steeplechase) and long-distance runners (5000 m, 10,000 m, marathon), whose training program included at least 5 h of physical training a day, every day;

2) (*n* = 18) Sedentary subjects who performed less than one hour of physical activity twice a week.

The following criteria defined the size and expected number of the experimental group: qualitative response model (baseline improvement yes/no); two parallel groups of 22 subjects each. Five subjects (one Athlete and four Sedentary) who were initially recruited for the trial were excluded from the final analysis because they withdrew before the end of the project.

All subjects were fully informed about the experimental protocols. The investigations were carried out following the 1975 Declaration of Helsinki guidelines (https://www.wma.net/what-we-do/medical-ethics/declaration-of-helsinki/), which were revised in Tokyo in 2004 and subsequently in 2013. All subjects approved and signed an informed consent document, authorizing the experimenter to use the results for scientific publication purposes; moreover, they each completed a four-day food diary. The Institutional Review Board at the University of Pavia approved the study. None of the subjects had taken any supplements or medications for 4 weeks prior to or during the experiment. All subjects were non-smokers, and none had taken any medication or drugs that could affect the results. The Athletes were all distance runners and cross-country track and field, who take part in national and international competitions. The inclusion criterion of Sedentary subjects was that they performed physical activity no more than twice a week, for a maximum of one hour each time.

### Materials

All chemicals and solvents used in this study were purchased from Sigma-Aldrich, St. Louis, MO, USA and J.T. Baker, USA. The DNA/RNA oxidative damage ELISA kit and the TNF-α ELISA kit were purchased from Cayman Chemical Company, Michigan, USA. Steroid Hormones ELISA kits were purchased from Diametra Diagnostic, Segrate-Milan, Italy. Software MetaDieta® for anthropometric and dietary/bromatological data was provided by METEDA srl. San Benedetto del Tronto (AP) Italy/EU. The blood samples were carried out at a Center of Clinical Biochemical Analysis, authorized by the National Health System (Pavia, Italy/EU).

Food Supplements (n-3) were soft-gel capsules containing fish oil (e.g. sardines, anchovies and mackerel, caught in open waters of the southern Pacific Ocean, Chile) at a minimum oil concentration of 40% EPA and 20% DHA in the form of ethyl esters. Each capsule of n-3 (1380 mg) contained 950 mg (> 95%) of fatty acids n-3 ethyl ester (EE), of which 400 mg EPA (43%) and 200 mg DHA (28%), and 2.5 mg of vitamin E (2500 ppm). The product was certified to be of high quality and it was free from doping substances with testosterone and nandrolone precursors, and from Beta2 agonists, diuretics, amphetamines and ephedrine.

### Experimental design

Before starting supplementation, anthropometric data (sex, age, weight, height, Body Mass Index-BMI) (Table 1) and a 4-day food diary were collected for each subject. All volunteer subjects participating in the study were instructed by a dietitian to fill in a food diary for 4 nonconsecutive days to evaluate energy intake and consumption of each nutrient, using the software MetaDieta® (METEDA S.r.l. – San Benedetto del Tronto, AP, Italy/EU). We focused on the intake values of n-6 and n-3 PUFAs and their ratio, as the dramatic change in eating habits in recent years has led to an imbalance in the ratio in favor of n-6 PUFAs, particularly in the Western diet [18]. This change has coincided with a worldwide increase in the incidence of inflammatory bowel disease (IBD) [19]. Some of the anti-inflammatory effects of n-3 PUFAs may be mediated by competition with n-6 PUFAs, because n-3 PUFAs act as a competitive substrate for the metabolism of n-6 PUFAs [20]. The data obtained by the software MetaDieta® were compared with the Dietary Reference Values (DRVs), particularly the Reference Intake range for macronutrients (RI), defined by Dietary Reference Intake for the Italian Population for specific ages: *IV Revisione dei Livelli di Assunzione di Riferimento di Nutrienti ed energia per la popolazione italiana* (LARN), guidelines containing information related to recommendations issued by Società Italiana di Nutrizione Umana (SINU) [21]. These values and recommendations were formulated according to the opinions of the European Food Safety Authority (EFSA) Panel on Dietetic Products, Nutrition and Allergies (NDA),published in 2010, which addressed the general principles for deriving and applying DRVs, an umbrella term for the complete set of nutrient reference values, including population reference intakes (PRIs), the average requirements (ARs), adequate intakes (AIs) and reference intake (RIs) ranges for macronutrients. These values indicate the amount of a nutrient which must be consumed on a regular basis to maintain health in an otherwise healthy individual (or population), referring to the European population [22]. In North America, the World Health Organization (WHO) recommends RI values of 6–10% of total energy for PUFA intakes for adults [23]. In the United States of America, The National Academies of Science Engineering Medicine recommends the following AI values: n-6, 17 g/day for young men and 12 g/day for young women; n-3, 1.6 and 1.1 g/day for men and women, respectively [24]. In Italy, the LARN recommends RI values of 4–8% of total energy from diet for n-6 PUFA and 0.5–2% of total energy from diet for n-3 PUFA for adults [21].

**Table 1:** Anthropometric data

	Age (years)	Weight (kg)	Height (m)	BMI (kg/m^**2**^)
**ATHLETE**				
Mean	25.56	60.06	1.74	19.87
SD	6.99	7.25	0.07	1.10
**SEDENTARY**				
Mean	22.86	57.86	1.70	19.85
SD	1.57	8.34	0.10	1.51

Anthropometric data for Athlete (n 21) and Sedentary (n 18) subjects

Data are expressed as mean values ± SD

For Athletes, the number of hours of daily training and the type of sport that were used to calculate energy consumption were also taken into consideration for this study.

The combination of all the above-mentioned parameters led us to the choice of an appropriate amount of daily supplementation: each subject took 4cps/day of n-3, i.e. about 4 g of n-3 fatty acids.

All subjects from both groups (Athletes and Sedentary) took 4cps/day of n-3 (2 at breakfast, 1 at lunch and 1 at dinner) for a period of 8 weeks. Compliance to the dosing regimen was monitored with the capsule count method: subjects received a fixed number of capsules and were asked to return any unused ones at their next follow-up visit.

In order to observe the association between long-chain n-3 PUFA supplementation, rich in EPA and DHA, and physical exercise, the variations in different markers of muscle workload, oxidative stress, steroid hormones as markers of exercise-induced stress, and inflammation, in Athletes and Sedentary subjects, were adopted as primary outcomes.

Our secondary outcome was to establish if a synergism existed between two stimuli: physical exercise and n-3 supplementation.

One sample of venous blood (red blood cells/RBC and plasma samples), one sample of saliva and one sample of urine were collected from all subjects at the following time points:

- before food supplementation (time T0) to evaluate different screening parameters;
- at the end of food supplementation (time T1), after 8 weeks (± 5 days), to evaluate the variations in all parameters.

The biological samples were collected in the morning, taking into account the circadian rhythm of the biochemical and physiological parameters, particularly of cortisol and testosterone. Blood samples taken from the antecubital vein were collected in BD Vacutainers Tubes (VacuLab® EDTA tubes). Samples were centrifuged (1000 x g for 10 min at 25 °C using centrifuge J6-MC by Beckman), and the resultant plasma was aliquoted and stored at − 80 °C. Urine samples were collected in sterile tubes and stored at − 80 °C. Saliva samples were collected in Salivette® (No. 51.1534.500, Sarsted). Samples were centrifuged (1000 x g for 2 min at 25 °C using centrifuge J6-MC by Beckman) to obtain a clear saliva sample ready to be stored at − 80 °C. All samples were analyzed in the same analytical session for each test using the same reagent lot.

### Markers of lipid profile/muscle workload/exercise-induced stress

The hematological parameters that were considered in the current pilot study were measured in a Healthcare structure Synlab® Pavia (Italy/EU). The parameters were the following: markers of lipid profile (Triglycerides, Total Cholesterol, HDL), markers of muscle workload (Creatinine, CPK, LDH, HGH, IGF-1) and markers of exercise-induced stress i.e. steroid hormones (Testosterone and Cortisol). Creatinine, a waste product from the normal breakdown of muscle tissue, was used as a test of kidney function [25]. Both CPK and LDH, which have important roles in cell energy processes, were used to assess damage to muscle tissues induced by injury [26]. We also assessed the blood concentrations of the polypeptide hormone HGH that enhances muscle mass, promotes lipolysis and gluconeogenesis, and stimulates the synthesis of IGF-1 in the liver. In addition to HGH, we measured IGF-1 because its synthesis can be altered by different factors, such as undernutrition, physical exercise status, stress levels, growth hormone insensitivity and lack of growth hormone receptors. IGF-1 enhances bone density, lean muscle mass, weight loss, carbohydrate metabolism and encourages cell regeneration and tissue repair [27].

### Markers of oxidative status and antioxidant parameters

Given that the best approach in assessing oxidative stress seems to be the evaluation of the balance between antioxidants and by-products of oxidative reactions in the organism [28], we decided to investigate markers of oxidative damage (Malondialdeyde (MDA), Protein Carbonyl content (PCc) and 8-hydroxy-2′-deoxyguanosine (8-OHdG) [29]) and markers of antioxidant ability (the enzyme activities of Glutathione peroxidase (GPx), Speroxide dismutase (SOD) and Catalase (CAT)). We also used the DPPH test to determine the free radical scavenging activity, by evaluating the reagent l,l’-diphenyl-2-picrylhydrazyl. The measurements were carried out in triplicate in the same laboratory. The mean of the three measurements was calculated and adopted. Their concentration and activities were assessed measuring absorbance by an UV-VIS spectrophotometer (Shimadzu UV1800) and a microplate spectrophotometer (BioTek ELx800).

MDA is one of the most reactive lipoperoxides ROOH (LPO), produced during the lipid peroxidation cascade of the PUFAs of the biological membranes, which are particularly susceptible to ROS-mediated oxidation due to their high double C=C bond content. The reactive and radical species has sufficient activity to steal a hydrogen atom from a lipid methyl group, inducing a lipid peroxidation cascade [30]. The main effect of lipid peroxidation on biological membranes is the overall decrease in their fluidity and severe damage to the membrane proteins [31]. Lipid peroxidation was investigated by quantifying MDA value in urine and plasma by the Erdelmeier method [32]. In this assay, two molecules of MDA react with N-methyl-2-phenilindole 10.3 mmol/l, at 45 °C and pH 3.6 for one hour to produce a stable complex that has maximum absorbance at λ 586 nm. The test was carried out in basal condition using the non-pretreated samples urine and plasma and in a stimulated condition using a pretreated sample of plasma with an oxidative agent, such as CuSO_4_ 0.5 mmol/l, at 37 °C for one hour.

PCc derived from protein carbonylation, an oxidation promoted by reactive oxygen species. It usually refers to a process that forms reactive ketones or aldehydes that are capable of reacting with the reagent 2,4-dinitrophenylhydrazine (DNPH) to form hydrazones. Protein oxidative damage involves both the loss of thiol groups and modifications to amino acids that constitute the polypeptide chain, in particular histidine. PCc is used as a marker of oxidative damage, which acts primarily on the side chains of the aminoacyl residues lysine, arginine, proline and threonine [33]. PCc was measured in RBC according to Levine and colleagues [34] after reaction with 2,4-Dinitrophenylhydrazine (DNPH) 10 mmol/l at 25 °C for one hour. Streptomycine sulfate 1% was used to decrease nucleic acid contamination. At the end of the reaction, the carbonyl content was calculated from the maximum absorbance (λ360–390 nm) and expressed as nmol on the amount of total proteins (mg), obtained applying the Lowry method [35].

ROS damage DNA either by an indirect mechanism or by direct interaction with molecules, forming 8-hydroxy-2′-deoxyguanosine (8-OHdG), a stable product derived from the attack of hydroxyl radical on guanine residues in the presence of oxygen or other oxidizing agents [36] and considered as a good biomarker of oxidative DNA damage [37]. The concentration in the urine was measured using the High Sensitivity-DNA/RNA oxidative Damage Elisa kit (Item № 589,320 Cayman Chemical, Michigan 48,108 USA) according to the manufacturer’s instructions (normal value: 10–3000 pg/ml). This immunoassay for the measure of DNA/RNA oxidative damage detects at λ405–420 nm all three oxidized guanine species: 8-hydroxy-2′-deoxyguanosine from DNA, 8-hydroxyguanosine from RNA, and 8-hydroxyguanine from either DNA or RNA.

The endogenous antioxidant capacity was evaluated by measuring antioxidant parameters such as the enzyme activities in blood (RBC) and the total free radical scavenging activity in saliva, applying colorimetric assays. GPx activity was determined following the formation of NADP+ according to the method of Flohe & Gunzler (1984) [38]. SOD activity was evaluated according to the capability of the samples to inhibit the reduction of cytochrome C with xanthine/xanthine oxidase, using the method developed by Flohe & Otting (1984) [39]. CAT activity was determined by measuring the decomposition of H_2_O_2_ into H_2_O, at 25 °C and pH 7.0, according to the method of Aebi (1984) [40]. The total free radical scavenging activity of low-molecular-weight nonenzymatic fraction (LMNEF) of whole saliva was determined by using the reagent l,l’-diphenyl-2-picrylhydrazyl (DPPH), using the method developed by Atsumi (1999) [41]. The DPPH is a relatively stable compound in alcoholic solution with a peak absorbance at λ 517 nm. The radical scavenging activity of the whole saliva was determined in terms of the decreasing rate of absorbance detected at 517 nm in a 40% ethanol-DPPH solution (pH 7.4) at room temperature. The free radical scavenging activity was calculated as [(A517 control - A517 sample) / A517 control], representing the concentration of DPPH scavenged for 1 ml of saliva.

### Markers of exercise-induced stress: steroid hormones in saliva

Testosterone is a steroid hormone secreted from the Leydig cells of the testes under hypothalamic and pituitary control defining the hypothalamo-pituitary-testicular (HPT) axis. It has both anabolic and anti-catabolic effects on muscle tissue [42]. Cortisol is a steroid hormone released from the adrenal cortex stimulated by an Adreno Corticotropic Hormone (ACTH); it is involved in the response to stress [43]. The ratio between the concentration of testosterone and cortisol (T/C) is frequently used as an indication of the level of exercise-induced stress. Alterations in the concentration of these hormones are responsible for modulating several responses induced by training, such as hypertrophy and strength gain. Regarding this aspect, the balance between these anabolic/catabolic hormones is often used as an overreaching index and as a predictive index of OTS [44]. In the current study, we measured these two hormones not only in the blood, but also in saliva. The latter was a non-invasive, stress-free alternative to serum, used for example to evaluate unbound steroids, such as testosterone and cortisol that show a correlation with their free form serum concentrations [45, 46]. The concentration of testosterone and cortisol in saliva was determined by a colorimetric competitive enzyme-linked immunosorbent assay (ELISA) method (Diametra Diagnostic S.r.l., Spello – Perugia, Italy/EU): testosterone saliva ELISA (Item № DKO021); cortisol saliva ELISA (Item № DKO020). The antigen in the sample competes with the antigenic testosterone or cortisol, conjugated with horseradish peroxidase (HRP) for binding to the limited number of antibodies anti testosterone or cortisol, coated on a 96-well plate. The colorimetric enzyme-linked immunosorbent reaction was detected at λ 450 nm by a microplate spectrophotometer (BioTek ELx800).

### Marker of inflammation

TNF-α is a 17 kDa polypeptide early mediator in the acute phase response of the inflammation process, with an important role in the initiation of the inflammatory cascade, including the induction of liver-produced acute phase proteins such as CRP, the activation and differentiation of monocytes and macrophages, the expression of major histocompatibility complex (MHC) class I and II, and the expression of adhesion molecules on endothelial cells [47]. The concentration of TNF-α in plasma was evaluated by an enzyme-linked immunosorbent assay (ELISA) method (Item № 589,201 Cayman Chemical, Michigan 48,108 USA), which gives TNF-α measurements within the range of 0–250 pg/ml, typically with a limit of detection of 1 pg/ml. The colorimetric enzyme-linked immunosorbent reaction was detected at λ405–420 nm by a microplate spectrophotometer (BioTek ELx800).

### Statistical analysis

Continuous variables were reported as mean values ± standard deviation (SD), and were analyzed using non-parametric tests (i.e., Friedman and Wilcoxon test, as appropriate), since these variables were not normally distributed (based on the Shapiro-Wilk statistic). All tests were two-sided and a *p*-value of less than 0.05 was considered as statistically significant. Since multiple measures were obtained from the same individuals, we used a Principal Component Analysis (PCA) to explore the relationships among the physiological parameters. The first 5 principal components (PC, all with eigenvalue > 1) which accounted for 60% of total variance and their scores were used as a set of independent variables in the subsequent analysis. In order to observe the consequences of n-3 supplementation in Athletes and Sedentary subjects, we followed the procedure described by Adams and Collyer (2009) [48]: firstly, we computed the vector of physiological change (size) as the difference among the five PC scores before and after supplementation; then, we estimated the difference in direction of physiological change of Athletes and Sedentary subjects as the angle among their vectors of physiological change. The significant differences in size and angle were calculated by two PERMANOVA (Permutational Multivariate Analysis of Variance) with 9999 permutations. Statistical analyses were performed using R ver. 3.2.2 (R Development Core Team, 2015) [48, 49].

For each parameter, two Mixed Models were applied: one contained the status (Athlete or Sedentary), supplementation and their interaction as fixed effects, and the identity of the individual as a random effect; and the other was the same as the first, but without the interaction between status and supplementation. To evaluate whether the expression of the parameters in Athletes before and after supplementation was different from that of Sedentary subjects before and after supplementation, i.e. whether the interaction between the factors was significant, the two models were compared with a likelihood Ratio Test.

Linear correlation between parameters was measured using the Pearson correlation coefficient (with R > 0.5 indicating a good correlation) and it was considered significant with values of t = 6.16, df = 8, *p*-value < 0.001.

## Results

The results of our study suggested that supplementation with n-3 PUFAs created a synergic variation in the parameters from a baseline state (T0) to a treated state after supplementation (T1), in terms of size and process, which was significantly different in Athletes compared to Sedentary subjects.

### Dietary analysis

The data obtained using a 4-day food diary analysis of n-3 and n-6 intake values for Athletes (Table 2) and Sedentary subjects (Table 3) were compared with one value (n-3: 1% of total energy intake; n-6: 6% of total energy intake) chosen from the RI values defined by the Italian LARN [21]. The obtained ratio n-6/n-3 for Athletes and Sedentary subjects was not different from the LARN value 6.00. However, both groups showed a lower intake of n-3 than the RI, even though the energy needs were sufficient.

**Table 2:** Dietary values for Athletes

	Energy needs	N-3 tot (over 4 days)	LARN n-3 (1%En)	N-6 tot (over 4 days)	LARN n-6 (6%En)	***RATIO*** (n-6/n-3)	LARN ***RATIO*** (n-6/n-3)
	**(Kcal)**	**(g)**	**(g)**	**(g)**	**(g)**	**(n-6/n-3)**	**(n-6/n-3)**
**mean**	2355	1.58	2.62	9.23	15.70	6.11	6.00
**SD**	505	1.02	0.56	6.93	3.37	1.89	0.00
***p value*** **n-3**		0.001					
***p value*** **n-6**				0.002			
***p value ratio*** **n-6/n-3**						0.827	
△ **n-3**		39.69%					
△ **n-6**				45%			

Data were obtained from a 4-day food diary, elaborated with the software MetaDieta®. The estimated values of Energy needs (kcal), amount of n-3 (g), n-6 (g) and their ratio n-6/n-3 were reported. The obtained data of n-6, n-3 and their ratio were compared with the value of Italian LARN reference (RI)

Data are expressed as mean values ± SD. Statistical analysis: repeated measures analysis of variance; level of significance: *p* < 0.05; The value of △ % was obtained by comparing values from the 4-day food diary with LARN values. Abbreviation: En, energy

**Table 3:** Dietary values for Sedentary subjects

	Energy needs	N-3 tot (over 4 days)	LARN n-3	N- 6 tot (over 4 days)	LARN n-6	RATIO (n-6/n-3)	LARN RATIO (n-6/n-3)
	**(kcal)**	**(g)**	**(g)**	**(g)**	**(g)**	**(n-6/n-3)**	**(n-6/n-3)**
**mean**	2183	1.32	2.43	5.26	14.56	4.50	6.00
**SD**	323	0.53	0.36	1.70	2.16	2.03	0.00
***p value*** **n-3**		0.009					
***p value*** **n-6**				0.001			
***p value*** **ratio n-6/n-3**						0.211	
△ **n-3**		46.17%					
△ **n-6**				31.88%			

Data were obtained from a 4-day food diary, elaborated with the software MetaDieta®. The estimated values of Energy needs (kcal), amount of n-3 (g), n-6 (g) and their ratio n-6/n-3 were reported. The obtained data of n-6, n-3 and their ratio were compared with the value of Italian LARN reference (RI)

Data are expressed as mean values ± SD. Statistical analysis: repeated measures analysis of variance; level of significance: p < 0.05; The value of △ % was obtained by comparing values from the 4-day food diary with LARN values. Abbreviation: En, energy

### Analysis of relationships among *parameters*

**Fig. 1:**
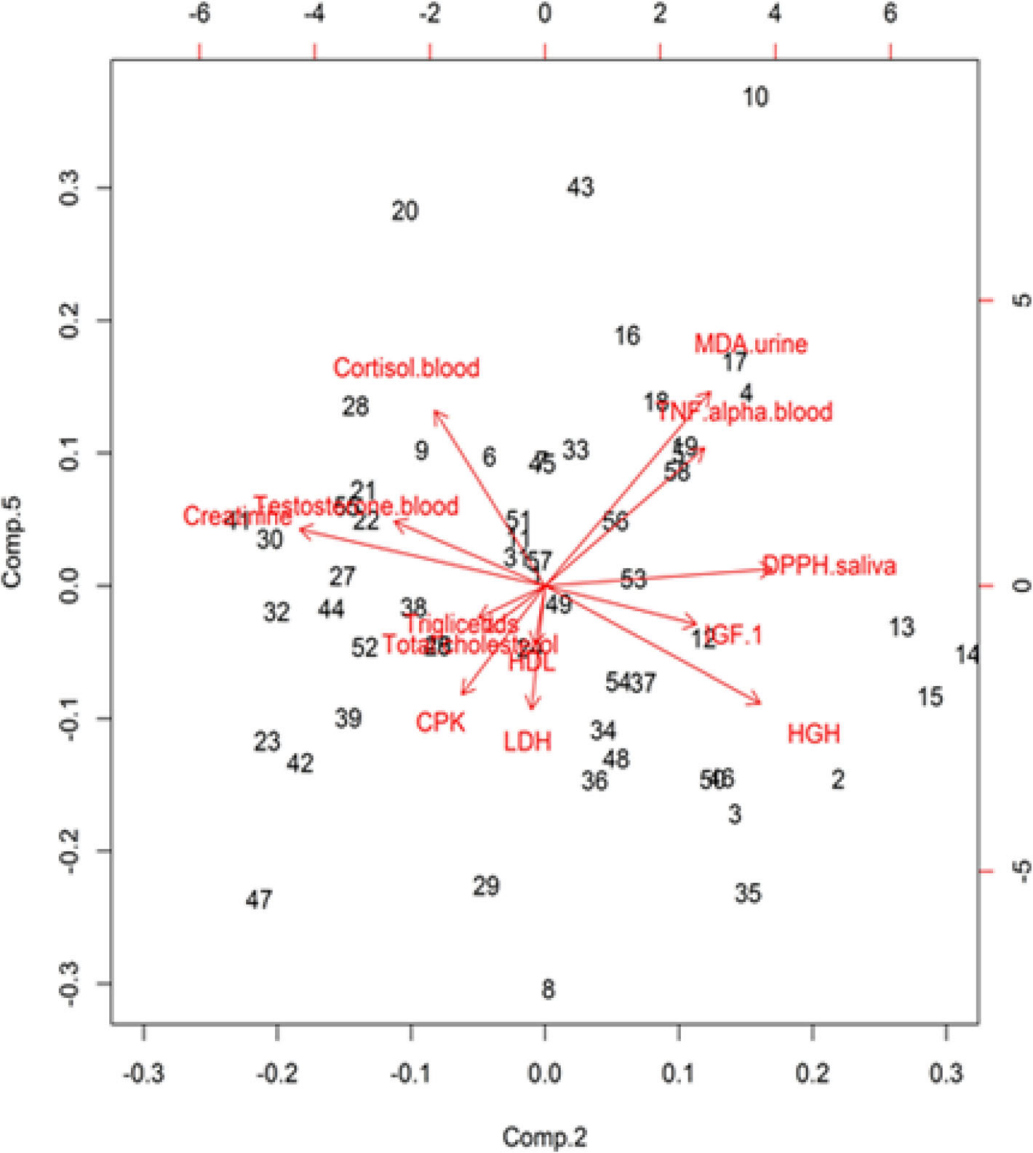
PCA graph. Components graph showing the two selected components (Comp 2 and Comp 5) and parameters

**Fig. 2:**
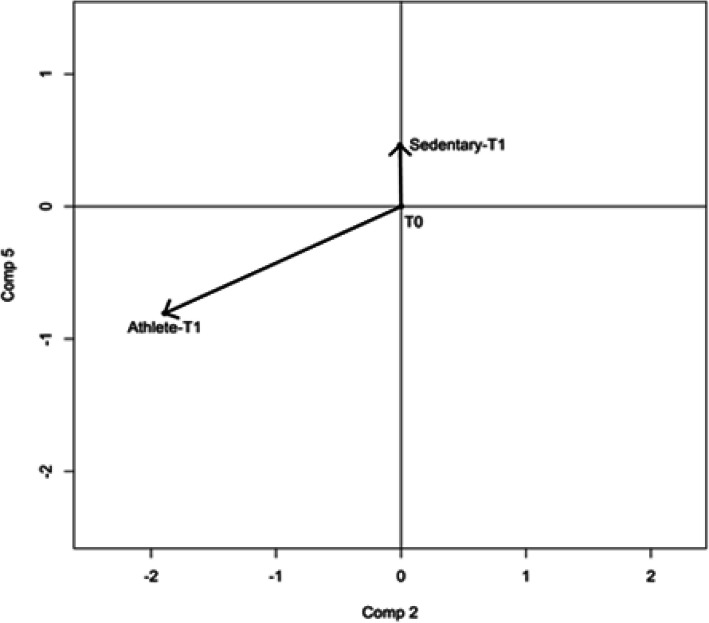
Multivariate Analysis of Variance. Difference in direction of physiological change of Athletes and Sedentary subjects as the angle between their vectors of physiological change considering Components (Comp) 2 and 5

Data were analyzed with PCA to explore the relationships among the parameters. The first 5 principal components (PC, all with eigenvalue > 1), accounting for 60% of total variance (Table 4), and their scores (Fig. 1) were used as a set of independent variables in the subsequent analysis. The principal components that seemed to explain most of the differences between Athletes and Sedentary subjects, before and after supplementation, were the 2nd and the 5th components (Figs. 1 and 2).

**Table 4:** Principal components

	Comp-1	Comp-2	Comp-3	Comp-4	Comp-5
**MDA-urine**	− 0.25	0.31*	− 0.04	0.02	0.51*
**DPPH-saliva**	− 0.19	0.43*	− 0.25	0.12	0.04
**Testosterone-blood plasma**	− 0.42	− 0.28	0.13	0.17	0.17
**Cortisol-blood plasma**	0.3	−0.21	− 0.05	0.12	0.46*
**TNF-***α***- blood plasma**	−0.22	0.3	−0.38	0.04	0.36*
**Creatinine-blood**	0.1	− 0.46*	−0.19	0.32	0.15
**Total cholesterol-blood**	0.17	−0.11	− 0.63	− 0.12	− 0.12
**Cholesterol-HDL-blood**	0.2	−0.02	− 0.39	0.56	− 0.16
**Tryglicerides-blood**	−0.01	− 0.13	− 0.3	−0.65	− 0.08
**CPK**	−0.46	− 0.16	0.03	0.21	−0.29
**LDH**	−0.44	− 0.03	− 0.18	0.07	− 0.33*
**HGH**	0.19	0.4*	−0.04	0.12	−0.31*
**IGF-1**	0.27	0.28	0.25	0.18	−0.1

Principal components (PC, all with eigenvalue > 1), accounting for 60% of total variance, and their scores. The values indicate how the biological parameters affect each component. The symbol (*) indicates the relevant effect on the component

### Variation in lipid profile and muscle workload

Considering Triglycerides, total Cholesterol and Cholesterol HDL, the mean values were inside the normal range indicated by laboratory standard references (Triglycerides 20–150 mg/dl; total cholesterol < 200 mg/dl; Cholesterol HDL > 40 mg/dl) for both groups (Table 5). Concerning triglycerides values, only the Athlete group showed a significant reduction after 8 weeks of n-3 supplementation (Table 5), but if we considered how biological parameters affected each component indicated in Table 4, we observed that total Cholesterol decreased more markedly in Athletes compared to Sedentary subjects.

**Table 5:** Lipid profile

		T0	SD	T1	SD	***p value***	△ %	
**Triglycerides (mg/dl)**	**ATHL**	82	34	64	22	2*10^*−4*^	21.95	**T1vsT0**
	**SED**	67	15	65	17	*0.35*	2.98	**T1vsT0**
						*0.26*	18.29	**T0**
						*0.90*	1.54	**T1**
**Total Cholesterol (mg/dl)**	**ATHL**	175	26	171	35	*0.47*	2.28	**T1vsT0**
	**SED**	180	18	184	21	*0.41*	2.17	**T1vsT0**
						*0.68*	2.78	**T0**
						*0.39*	7.06	**T1**
**HDL (mg/dl)**	**ATHL**	50	9	50	11	*1.00*	0.00	**T1vsT0**
	**SED**	54	8	55	9	*0.76*	1.82	**T1vsT0**
						*0.21*	8.09	**T0**
						*0.36*	9.10	**T1**

Data are expressed as mean values ± SD comparing two groups and two periods. Statistical analysis: repeated measures analysis of variance; level of significance: p < 0.05;△ % for the difference between mean values, T1vs T0 intra-group, Sedentary (SED) vs Athlete (ATHL) groups for T0 and T1

Regarding muscle workload, the Athletes showed a significant increase in creatinine after 8 weeks of supplementation (Table 6) (Laboratory standard references: male 0.70–1.30 mg/dl; female 0.44–1.00 mg/dl); this could be due to the protein-rich diet of the Athletes and to the physical exercise workload. On the contrary, CPK (standard laboratory reference indicates an optimal CPK value < 200 U/l in the blood) and LDH (standard laboratory reference indicates optimal LDH values: 125–243 U/l) results showed that there were no significant variations before or after supplementation in either group (Athletes or Sedentary subjects), as shown in Table 6, but the mean values of the Athletes were higher than those of the Sedentary subjects for both parameters (Table 6). Pearson correlation coefficient (R = 0.86) indicated a high correlation between CPK and LDH in blood (Table 7).

**Table 6:** Muscle workload

		T0	SD	T1	SD	***p value***	△ %	
**Creatinine (mg/dl)**	**ATHL**	0.730	0.068	0.850	0.077	8*10^*−4*^	14.12	**T1vsT0**
	**SED**	0.840	0.131	0.790	0.098	*0.08*	5.95	**T1vsT0**
						*0.23*	13.09	**T0**
						*0.31*	7.06	**T1**
**CPK (U/l)**	**ATHL**	187	124	198	115	*0.76*	5.56	**T1vsT0**
	**SED**	56	21	73	39	*0.11*	23.29	**T1vsT0**
						*0.02*	70.05	**T0**
						*0.02*	63.13	**T1**
**LDH (U/l)**	**ATHL**	179	38	175	33	*0.70*	2.23	**T1vsT0**
	**SED**	152	50	139	30	*0.30*	8.55	**T1vsT0**
						*0.16*	15.08	**T0**
						*0.02*	20.57	**T1**

Data are expressed as mean values ± SD comparing two groups and two periods. Statistical analysis: repeated measures analysis of variance; level of significance: p < 0.05;△ % for the difference between mean values, T1vs T0 intra-group, Sedentary (SED) vs Athlete (ATHL) groups for T0 and T1

**Table 7:** Analysis of Correlation

	MDA urn	DPPH slv	T slv	C slv	T/C slv	T bld	C bld	T/C bld	TNFα bld	Creat	Cholest TOT	Cholest HDL	Trigly	CPK	LDH	HGH	IGF-1
MDAurn	1.00	0.32	−0.12	0.75	−0.44	−0.06	0.56	−0.04	−0.12	−0.37	0.48	−0.26	0.82	−0.01	0.28	0.12	−0.39
DPPHslv	0.32	1.00	0.04	−0.02	0.02	0.09	−0.26	0.29	0.13	−0.14	−0.37	−0.23	0.42	0.38	0.57	0.04	−0.28
T_slv	−0.12	0.04	1.00	−0.20	0.67	0.91	−0.04	0.73	−0.40	0.48	−0.04	0.02	−0.18	0.59	0.56	−0.80	−0.19
C_slv	0.75	−0.02	−0.20	1.00	−0.77	− 0.26	0.90	− 0.48	−0.19	− 0.48	0.51	− 0.07	0.45	− 0.53	−0.26	0.13	0.08
T/C slv	−0.44	0.02	0.67	−0.77	1.00	0.72	−0.63	0.81	−0.17	0.52	−0.30	−0.08	− 0.30	0.85	0.58	−0.42	− 0.30
T_bld	−0.06	0.09	0.91*	−0.26	0.72	1.00	−0.16	0.88	−0.47	0.43	0.04	−0.11	−0.10	0.74	0.71	−0.76	−0.49
C_bld	0.56	−0.26	−0.04	0.90	−0.63	− 0.16	1.00	− 0.51	−0.05	− 0.13	0.71	0.17	0.17	−0.56	−0.42	0.00	0.25
T/C bld	−0.04	0.29	0.73	−0.48	0.81	0.88	−0.51	1.00	−0.40	0.28	−0.22	−0.28	0.12	0.91	0.91	−0.56	−0.67
TNFαbld	−0.12	0.13	−0.40	−0.19	− 0.17	−0.47	− 0.05	−0.40	1.00	0.37	0.09	0.14	0.03	−0.29	−0.36	0.30	0.18
Creat	−0.37	−0.14	0.48	−0.48	0.52	0.43	−0.13	0.28	0.37	1.00	0.27	0.48	−0.51	0.27	0.06	−0.58	0.07
Cholest TOT	0.48	−0.37	−0.04	0.51	−0.30	0.04	0.71	−0.22	0.09	0.27	1.00	0.39	0.10	−0.27	−0.21	− 0.04	−0.05
Cholest HDL	−0.26	−0.23	0.02	−0.07	− 0.08	−0.11	0.17	−0.28	0.14	0.48	0.39	1.00	−0.57	−0.33	− 0.36	−0.01	0.66
Trigly	0.82	0.42	−0.18	0.45	−0.30	−0.10	0.17	0.12	0.03	−0.51	0.10	−0.57	1.00	0.09	0.43	0.23	−0.59
CPK	−0.01	0.38	0.59	−0.53	0.85	0.74	−0.56	0.91	−0.29	0.27	−0.27	−0.33	0.09	1.00	0.86	−0.38	−0.60
LDH	0.28	0.57	0.56	−0.26	0.58	0.71	−0.42	0.91	−0.36	0.06	−0.21	−0.36	0.43	0.86	1.00	−0.39	−0.73
HGH	0.12	0.04	−0.80	0.13	−0.42	−0.76	0.00	−0.56	0.30	−0.58	− 0.04	−0.01	0.23	−0.38	− 0.39	1.00	0.26
IGF-1	−0.39	−0.28	− 0.19	0.08	− 0.30	−0.49	0.25	−0.67	0.18	0.07	−0.05	0.66	−0.59	−0.60	− 0.73	0.26	1.00

The high values (R > 0.5) indicate a significant correlation between the analyzed parameters. Abbreviations: *bld* blood; *C* Cortisol; *Cholest* Cholesterol; *Creat* Creatinine; *slv* saliva; *T* Testosterone; *T/C* RATIO T/C; *Trigly* Triglyceride; *urn* urine

The results showed that there were no significant differences among values before supplementation, and no variations in values after supplementation in either group (Athletes or Sedentary subjects) for both biomarkers HGH and IGF-1 (Table 8), which had optimal HGH standard laboratory reference values: male < 3.00, female < 6.00 ng/ml; IGF-1 values (16–24 years old): 150–480 ng/ml.

**Table 8:** Muscle Workload

		T0	SD	T1	SD	***p value***	△ %
**HGH (ng/ml)**	**ATHL**	2.9	4.5	2.6	3.6	*0.86*	10.34 **T1vsT0**
	**SED**	4.5	5.8	5.6	4.4	*0.54*	19.64 **T1vsT0**
						*0.46*	35.56 **T0**
						*0.09*	53.57 **T1**
**IGF-1 (ng/ml)**	**ATHL**	290	142	306	115	*0.68*	5.23 **T1vsT0**
	**SED**	271	78	274	81	*0.82*	1.09 **T1vsT0**
						*0.76*	6.55 **T0**
						*0.65*	10.46 **T1**

Data are expressed as mean values ± SD comparing two groups and two periods. Statistical analysis: repeated measures analysis of variance; level of significance: p < 0.05;△ % for the difference between mean values, T1vs T0 intra-group, Sedentary (SED) vs Athlete (ATHL) groups for T0 and T1

### Variations in the markers of oxidative status and antioxidant parameters

**Fig. 3:**
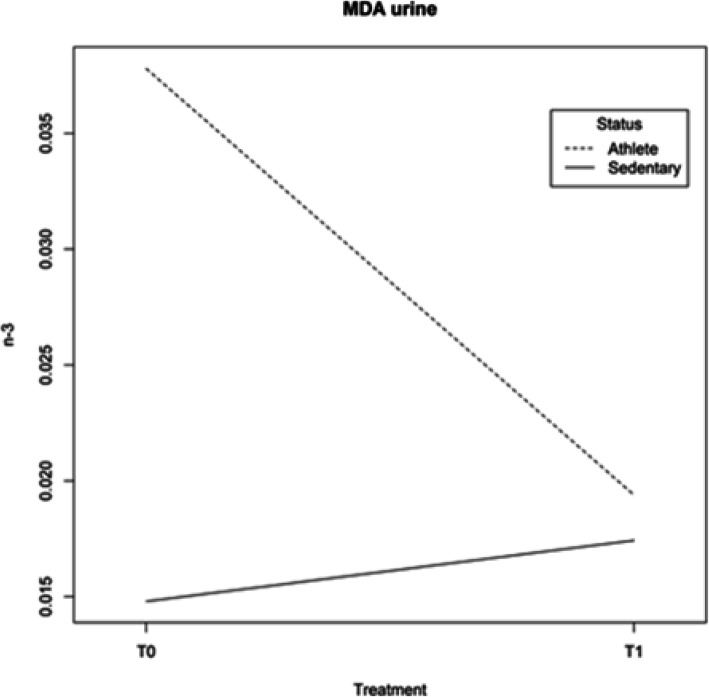
MDA in urine for Athletes and Sedentary subjects in different periods: before (T0) and after (T1) supplementation. Interactions between factors are cogent and significant (L ratio = 6.095124; *p* value = 0.0136)

MDA. The n-3 supplementation induced a significant reduction in the lipid peroxidation marker (MDA) after 8 weeks of treatment in plasma for both Athletes and Sedentary subjects, whereas, the removed metabolite in urine showed a significant reduced value only in Athletes after supplementation and not in Sedentary subjects (Table 9). Moreover, MDA decreased more markedly in Athletes than in Sedentary subjects, showing that the supplementation had different effects depending on the status (Athletes or Sedentary subjects) (Fig. 3).

**Table 9:** Oxidative status: MDA, PCc, 8-OHdG

		T0	SD	T1	SD	***p value***	△ %	
**MDA URINE (**μ**mol/ml)**	**ATHL**	0.030	0.017	0.019	0.011	*0.02*	36.67	**T1vsT0**
	**SED**	0.016	0.02	0.017	0.011	*0.45*	5.88	**T1vsT0**
						*0.10*	46.67	**T0**
						*0.70*	10.53	**T1**
**MDA BASAL PLASMA (**μ**mol/ml)**	**ATHL**	0.026	0.015	0.002	0.002	1*10^*−6*^	90.59	**T1vsT0**
	**SED**	0.003	0.002	0.001	0.001	*0.01*	58.82	**T1vsT0**
						6*10^*−4*^	86.67	**T0**
						*0.30*	41.67	**T1**
**MDA STIMULATED PLASMA (**μ**mol/ml)**	**ATHL**	0.053	0.017	0.016	0.003	7.0*10^*−8*^	69.19	**T1vsT0**
	**SED**	0.018	0.002	0.015	0.002	1*10^*−3*^	18.03	**T1vsT0**
						2.7*10^*−5*^	65.41	**T0**
						*0.30*	7.98	**T1**
**PCc (nmol/mg)**	**ATHL**	3.83	2.85	3.73	2.32	*0.92*	2.71	**T1vsT0**
	**SED**	3.50	2.12	5.31	1.61	*0.1*	34.01	**T1vsT0**
						*0.76*	8.61	**T0**
						*0.09*	29.75	**T1**
**8-OHdG (ng/ml)**	**ATHL**	27.87	22.50	56.47	45.60	*0.11*	50.65	**T1vsT0**
	**SED**	73.77	75.00	67.32	63.90	*0.87*	8.74	**T1vsT0**
						*0.2*	62.36	**T0**
						*0.72*	16.12	**T1**

Data are expressed as mean values ± SD comparing two groups and two periods. Statistical analysis: repeated measures analysis of variance; level of significance: p < 0.05;△ % for the difference between mean values, T1vs T0 intra-group, Sedentary (SED) vs Athlete (ATHL) groups for T0 and T1

PCc. Even though proteins are easy targets of oxidative modifications as induced by ROS and lipid peroxidation products (MDA and HNE), oxidized proteins are harmful to the maintenance of cellular homeostasis as they require rapid removal by proteolytic digestion [33, 34]; indeed, our results, as reported in Table 9, did not show differences between groups and variation of the PCc values after 8-weeks of supplementation, suggesting that neither physical exercise nor n-3 PUFA supplementation could affect protein carbonylation.

8-OHdG. This parameter of oxidative damage on DNA, such as PCc, did not show significant differences between values before and after treatment of n-3 supplementation for 8 weeks (Table 9).

**Fig. 4:**
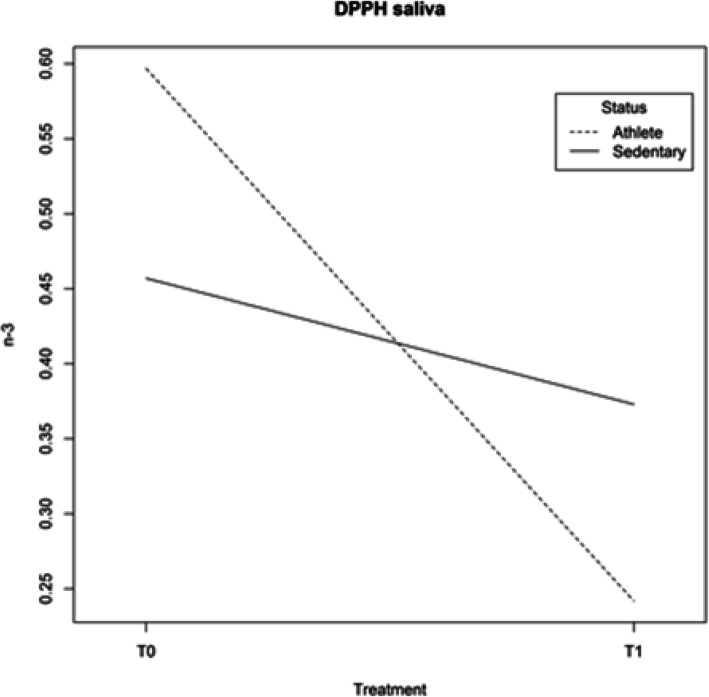
DPPH in saliva for Athletes and Sedentary subjects in different periods: before (T0) and after (T1) supplementation. Interactions between factors are cogent and significant (L ratio = 15.37332; p value = 1e-04)

The enzymatic antioxidant activities of GPx and CAT, which convert H_2_O_2_ to H_2_O, increased after 8 weeks of supplementation in both groups: Athletes and Sedentary subjects (Table 10). However, the supplementation did not affect SOD activity, which promoted the dismutation of superoxide into H_2_O_2_ (Table 10). Regarding the antioxidant capacity evaluated as DPPH scavenging in saliva, the results, reported as the concentration of DPPH (μmol) scavenged by 1 ml of saliva, showed a decrease in the total free radical scavenging activity of LMNEF of whole saliva. The DPPH value decreased more in Athletes than in Sedentary subjects (Table 10, Fig. 4), suggesting that the supplementation had a negative effect on the scavenging activity of saliva and that the effects were different in the two groups (Athletes or Sedentary subjects) (Fig. 4).

**Table 10:** Antioxidant parameters: GPx, SOD, CAT, DPPH

		T0	SD	T1	SD	*p value*	△ %	
**GPx** **(U/gHb)**	**ATHL**	9.78	2.00	13.70	3.39	6.2*10^*−4*^	28.61	**T1vsT0**
	**SED**	6.55	2.97	17.99	4.55	4.8*10^*−4*^	63.61	**T1vsT0**
						4*10^*−3*^	33.08	**T0**
						1.7*10^*−3*^	23.83	**T1**
**SOD** **(U/gHb)**	**ATHL**	722	180	761	129	*0.24*	5.05	**T1vsT0**
	**SED**	598	176	692	65	*0.07*	13.68	**T1vsT0**
						*0.13*	17.26	**T0**
						*0.19*	8.95	**T1**
**CAT (mmol/min)**	**ATHL**	0.007	0.002	0.010	0.006	3*10^*−3*^	38.68	**T1vsT0**
	**SED**	0.007	0.002	0.028	0.008	2*10^*−4*^	76.49	**T1vsT0**
						*0.76*	2.98	**T0**
						2*10^*−6*^	62.81	**T1**
**DPPH** **(**μ**mol/ml)**	**ATHL**	0.589	0.102	0.242	0.173	1.4*10^*−7*^	58.91	**T1vsT0**
	**SED**	0.457	0.047	0.373	0.067	*0.02*	18.38	**T1vsT0**
						3*10^*−3*^	22.41	**T0**
						*0.07*	35.12	**T1**

Data are expressed as mean values ± SD comparing two groups and two periods. Statistical analysis: repeated measures analysis of variance; level of significance: p < 0.05;△ % for the difference between mean values, T1vs T0 intra-group, Sedentary (SED) vs Athlete (ATHL) groups for T0 and T1

### Variation in exercise-induced stress markers

**Fig. 5:**
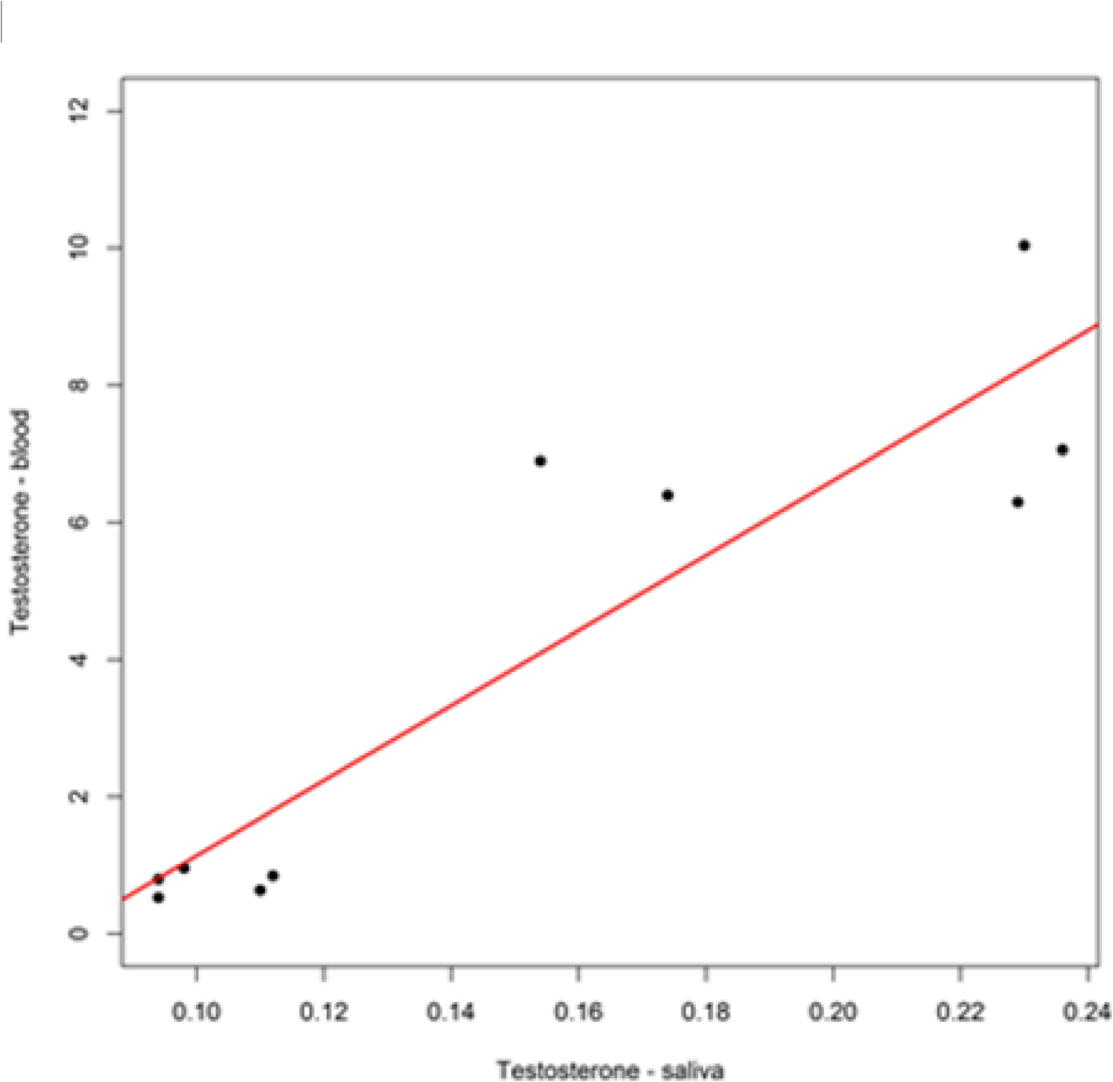
Linear correlation between Testosterone measured in the blood and in saliva. Testosterone: Pearson correlation coefficient (R = 0.91) indicates a high correlation. Pearson correlation test is significant (t = 6.16, df = 8, P-value < 0.001)

**Fig. 6:**
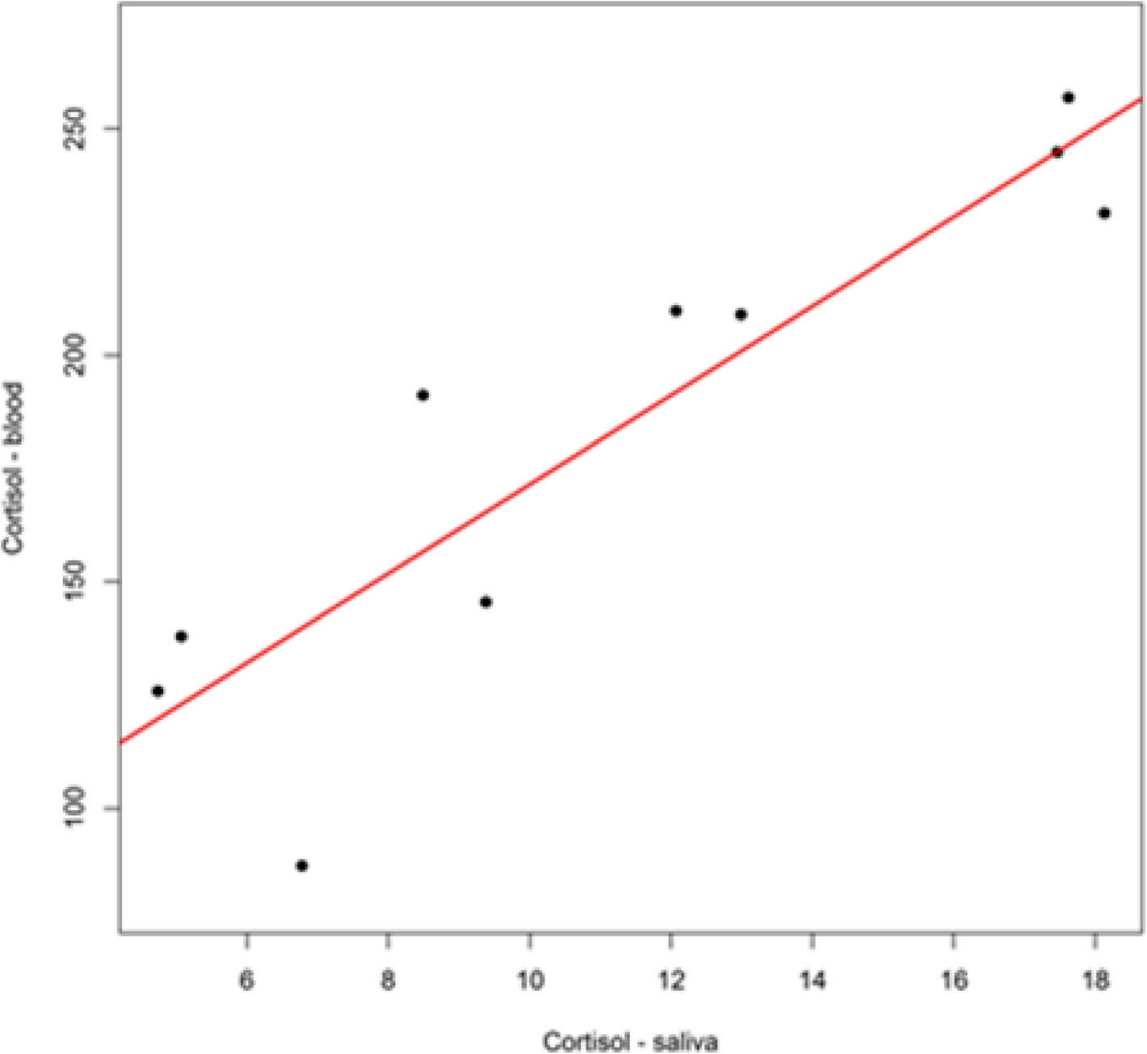
Linear correlation between Cortisol measured in the blood and in saliva. Cortisol: Pearson correlation coefficient (R = 0.90) indicates a high correlation. Pearson correlation test is significant (t = 5.83, df = 8, *P*-value < 0.001)

There was a multivariate significant difference between Athletes and Sedentary subjects: Cortisol tended to decrease in Athletes, whereas it increased in Sedentary subjects after supplementation (Table 11). Pearson correlation coefficient (R = 0.91) indicated a high correlation for Testosterone in blood and saliva (Fig. 5). The same result was obtained for Cortisol, for which Pearson correlation coefficient (R = 0.90) indicated a high correlation in blood and saliva (Fig. 6).

**Table 11:** Exercise-induced stress levels: Testosterone and Cortisol

		T0	SD	T1	SD	*p value*	△ %	
**TOTAL TESTOSTERONE Plasma** **(ng/ml)**	**ATHL**	4.8	3.21	4.67	2.84	*0.34*	1.67	**T1vsT0**
	**SED**	2.53	3.59	2.19	2.72	*0.26*	13.44	**T1vsT0**
						*0.34*	47.29	**T0**
						*0.26*	53.10	**T1**
**TESTOSTERONE** **Saliva** **(pg/ml)**	**ATHL**	151	38	139	49	*0.16*	7.88	**T1vsT0**
	**SED**	108	57	113	41	*0.41*	4.17	**T1vsT0**
						*0.10*	28.56	**T0**
						*0.28*	19.07	**T1**
**CORTISOL** **Plasma** **(ng/ml)**	**ATHL**	160	53	169	25	*0.22*	5.83	**T1vsT0**
	**SED**	176	35	216	44	*0.01*	18.43	**T1vsT0**
						*0.46*	9.37	**T0**
						3*10^*−3*^	21.50	**T1**
**CORTISOL** **Saliva** **(ng/ml)**	**ATHL**	11.11	5.12	8.54	1.06	*0.15*	23.13	**T1vsT0**
	**SED**	7.57	3.43	12.65	3.53	*0.01*	40.16	**T1vsT0**
						*0.28*	31.86	**T0**
						*0.04*	32.49	**T1**

Data are expressed as mean values ± SD comparing two groups and two periods. Statistical analysis: repeated measures analysis of variance; level of significance: p < 0.05;△ % for the difference between mean values, T1vs T0 intra-group, Sedentary (SED) vs Athlete (ATHL) groups for T0 and T1

**Fig. 7:**
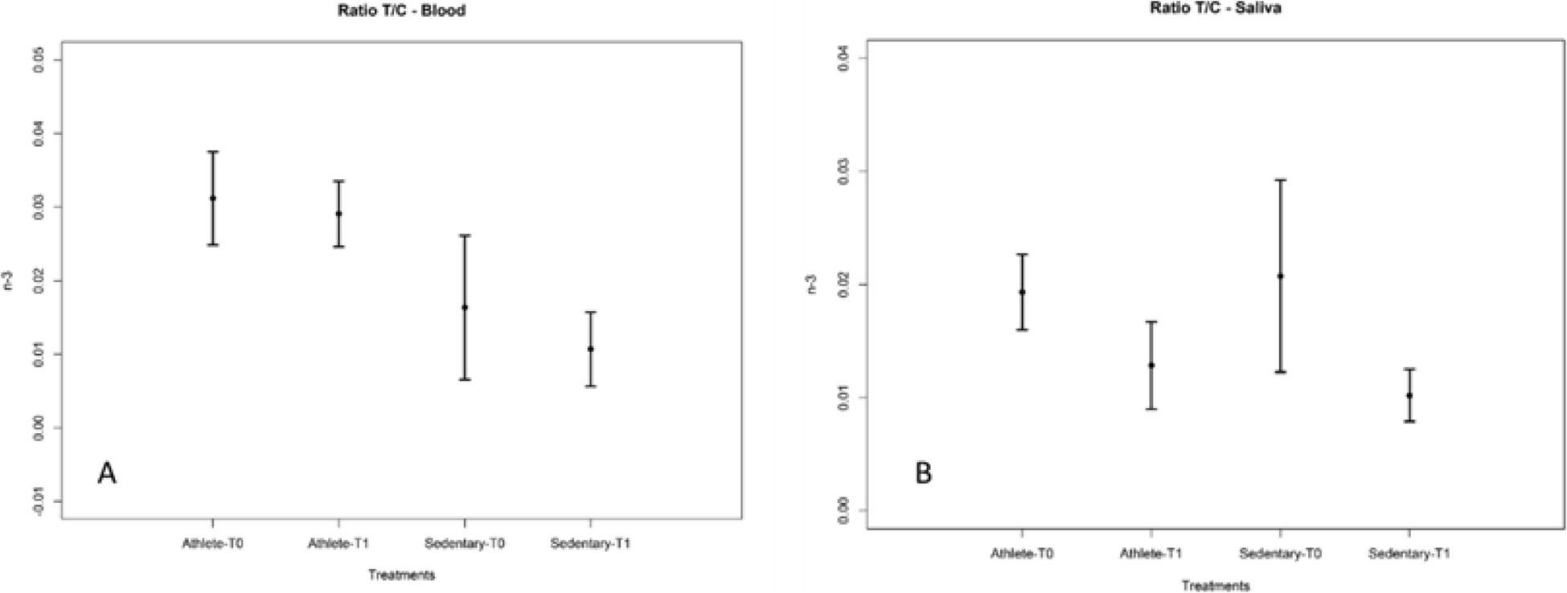
T/C ratio in plasma blood (A) and saliva (B) for Athletes and Sedentary subjects in different periods: before (T0) and after (T1) supplementation. Data are expressed as mean ± SD

Moreover, the correlation between parameters (Table 7) was evaluated, resulting in the following findings: 1) a strong correlation between the T/C ratio in blood and the T/C ratio in saliva with parameters from which they were generated; 2) a strong correlation between Testosterone in blood and in saliva (Fig. 5); 3) a strong correlation between Cortisol in blood and in saliva (Fig. 6). To maximize the diversity of parameters for statistical analysis, Testosterone and Cortisol measured in saliva and the two reports “ratio T/C “ were eliminated. Moreover, the T/C ratio values in plasma blood and saliva for both groups was reported in Fig. 7. The T/C ratio tended to decrease after supplementation in both groups because Cortisol increased, but the variation was more evident in Sedentary subjects than in Athletes.

### Variation in the marker of inflammation

**Fig. 8:**
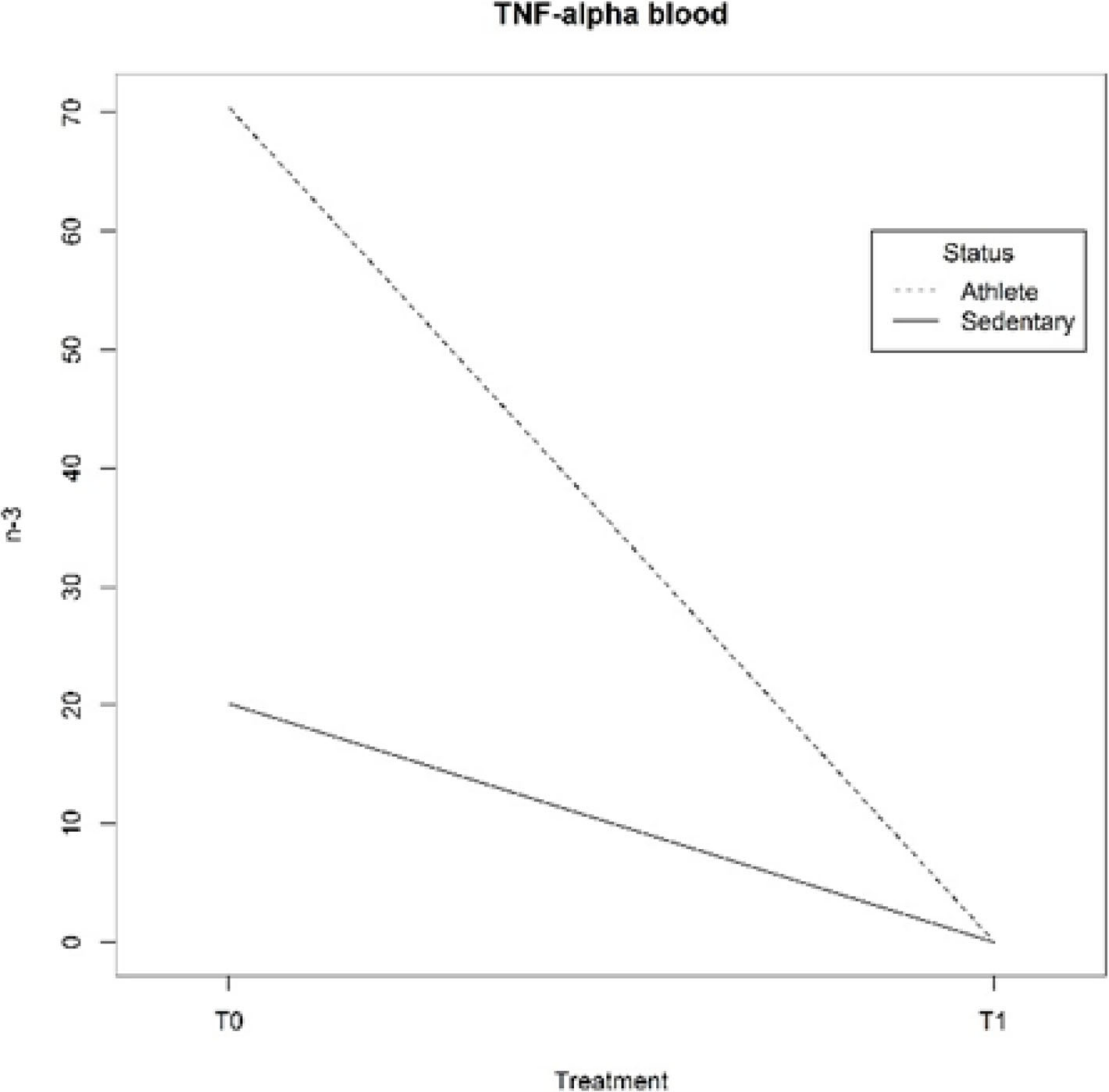
TNF-α in plasma blood for Athletes and Sedentary subjects in different periods: before (T0) and after (T1) supplementation. Interactions between factors are cogent and significant (model MCMCglmm 106 iterations with a burn-in of 10,000 iterations and a thinning interval of 100)

Regarding the results of the inflammatory marker, the plasma concentration of TNF-α decreased more evidently in Athletes compared to Sedentary subjects (Table 12). The supplementation had different effects depending on the status (Athletes or Sedentary subjects), as reported in Fig. 8.

**Table 12:** Inflammatory marker: TNF-α

		T0	SD	T1	SD	*p value*	△ %	
**TNF-**α **(pg/ml)**	**ATHL**	54.57	64.80	0.05	18.80	*0.02*	99.91	**T1vsT0**
	**SED**	18.18	0.20	0.00	0.00	*0.01*	100.00	**T1vsT0**
						*0.24*	66.68	**T0**
						*0.52*	100.00	**T1**

Data are expressed as mean values ± SD comparing two groups and two periods. Statistical analysis: repeated measures analysis of variance; level of significance: p < 0.05;△ % for the difference between mean values, T1vs T0 intra-group, Sedentary (SED) vs Athlete (ATHL) groups for T0 and T1

## Discussion

The main difference between the two examined groups (Athletes and Sedentary subjects) before supplementation with about 4 g/day of n-3 PUFAs, rich in EPA and DHA, for 8 weeks, was their physical exercise status. The results of the current study suggested that the n-3 supplementation created a synergic variation in the parameters from a baseline state (T0) to a treated state after supplementation (T1), in terms of size and modality, which was significantly different in Athletes compared to Sedentary subjects.

It is well known that intense and prolonged exercise is characterized by a large formation of radical oxygen and nitrogen compounds, as well as by an imbalance of homeostasis between pro-oxidant/anti-oxidant species and a general increase in the inflammatory state of the human body [2–5]. In this pilot study, the analysis of the results, obtained after n-3 fatty acid supplementation of about 4 g/day for 8 weeks, highlighted some benefits in different areas. In particular, n-3 PUFA supplementation may affect oxidative stress, as suggested by the significant decrease in MDA, a marker of oxidative stress [28], and by the significant increase in enzyme antioxidant activities after supplementation. MDA decreased in Athletes more than in Sedentary subjects, suggesting that the treatment had different effects depending on the amount of exercise performed (Athletes or Sedentary subjects). On the contrary, the supplementation had no effect on the other markers of oxidative damage, such as carbonylated proteins and 8-OHdG. Therefore, these results lead us to believe that the localization of n-3, extracellular and trans membrane EPA and DHA, which act on phospholipids, may influence the activity of these fatty acids. Our study also showed promising results for enzymatic antioxidant capacity: supplementation was shown to improve the activity of GPx and CAT both in Athletes and Sedentary subjects.

Another important result was obtained regarding inflammation, namely that the consumption of about 4 g/day of n-3 PUFAs for 8 weeks was shown to reduce the concentration of pro-inflammatory cytokine TNF-α, both in Athletes and Sedentary subjects, but TNF-α decreased more evidently in Athletes than in Sedentary subjects. This result leads us to hypothesize positive effects of n-3 supplementation on an inflammatory status, but the high variability of the data collected for Athletes (time T0) suggests the need to use a larger sample size than we used in this pilot study.

Moreover, the current investigation demonstrated that a linear correlation existed between the steroid hormones Testosterone and Cortisol measured in saliva and blood, according to literature data [50], validating the use of saliva samples as an innovative and non-invasive matrix instead of blood samples. The use of saliva samples was simple, quick, cheap, non-invasive and could be reproduced various times a day; these characteristics were very important considering a study involving Athletes because it was possible to take saliva samples during different competitions. As saliva samples are easily accessible, they can be used to assess competition or training conditions and for anti-doping purposes. The results of this pilot study showed that Cortisol significantly increased in the Sedentary group after the supplementation period. There was a multivariate significant difference between Athletes and Sedentary subjects: Cortisol tended to decrease in Athletes, but increase in Sedentary subjects after supplementation. This hormone, known as the stress hormone, seemed to be more susceptible to change in Sedentary subjects than in Athletes. This observation could be due to the different lifestyle Sedentary subjects have compared to Athletes: the latter group have more stable hormone levels than the former group because of their daily workouts and more regular way of life. In this case, the increased Cortisol in Sedentary subjects could not be due to the n-3 supplementation because this increase was not found in Athletes either. It is likely that the effect of n-3 supplementation in Athletes is more evident because it is sustained by their ability to control their Cortisol level, which dramatically increases in agonist athletes, who are subjected to physical stress [43, 44]. The ratio values did not indicate that subjects overreached, indeed all values were higher than 0.00035 and there was no decrease in ratio (> 30%), as reported by Adlercreutz and colleagues [51]. It is known that the hypothalamo-pituitary-adrenal (HPA) axis, which controls cortisol release, plays an important role in the adaptation to endurance training and acute response to exercise. Cortisol exerts catabolic effects on muscle tissue [43] and has important metabolic functions, such as influencing the metabolism of lipids, proteins and glucose. It increases the mobilization of fatty acids from fat reserves to active tissue and raises blood glucose [52]. Intense physical exercise increases Cortisol [53], which may inhibit protein synthesis with consequent increase in muscle mass by its catabolic effect [52].

According to the obtained dietary data, all subjects (Athletes and Sedentary subjects) showed a lower intake of n-3 PUFAs than the recommended intake by LARN 2014 [21]. After supplementation, the mobilization of fatty acids was further improved, as shown by the reduction in triglycerides and total cholesterol levels in the Athletes’ blood. This effect was more evident in Athletes than in Sedentary subjects; on the contrary, there were no changes in HDL levels in either experimental group. It is important to point out that all voluntary subjects were young people (< 35 years old), who showed basal lipid values that were within the normal standard range, so it would be interesting to plan a new study considering subjects with high cholesterol levels.

The Athlete group showed a significant increase in the creatinine value after 8 weeks of supplementation; this could be due to the Athletes’ protein-rich diet. Moreover, the muscular work carried out by the Athlete group leads to high levels of LDH and CPK, as well as an accentuated oxidative status, particularly in Athletes, confirmed by the levels of MDA in plasma and urine that proved to be higher than reported in literature [54]. The study showed that there were no significant variations in muscle workload markers (CPK, LDH and growth hormones such as HGH and IGF-1) after n-3 PUFA supplementation in both experimental groups.

In summary, our pilot study suggested that strenuous physical exercise training leads to increased triglyceride levels, CPK, LDH, MDA, TNF-α and Gpx (not balanced by CAT increase), thus the endogenous antioxidant defenses are not sufficient to counteract oxidative stress (increased risk of overtraining syndrome).

Dietary supplementation with n-3 PUFAs (4 g/day for 8 weeks), enriched in EPA and DHA, decreased triglyceride levels, MDA and TNF-α and increased Gpx and CAT. Therefore, it may partially counteract oxidative stress, protecting middle- and long-distance running Athletes from the risk of overtraining.

Overall, our study showed a marked effect of n-3 supplementation in Athletes compared to Sedentary subjects; this effect may be due to an adaptation “hormesis” to stress conditions in athletes [55], expressed by a high threshold antioxidant capacity, contrasting oxidative status. Therefore, an interaction seemed to exist between two stimuli, physical exercise and n-3 supplementation, on the evaluated markers.

For the future, it would be interesting to enlarge the inflammatory panel to include other inflammatory markers, such as interleukins (IL) IL-6 and IL-8. Moreover, it would be interesting to include a third experimental group of Athletes who practice regular but non-strenuous physical exercise. Furthermore, considering that running-specific studies are scarce, this study increases the data available in scientific literature. Even though the data were obtained from a small sample, the results of the current study could highlight recommendations for future research to optimize running performance with nutritional interventions.

## Conclusions

n-3 PUFA supplementation may be useful as a nutritional countermeasure to strenuous exercise-induced oxidative stress and inflammation in Athletes, but further in vivo studies in humans need to be carried out.

## Data Availability

The datasets used and/or analyzed during the current study and not shown in the paper are available from the corresponding author on reasonable request.

## Abbreviations

AA: Arachidonic acid
ACTH: Adreno corticotropic hormone
AIs: Adequate intakes
AP: Ascoli Piceno
ARs: Average requirements
ATHL: Athletes
bld: blood
BMI: Body mass index
C: Cortisol
CAT: Catalase
Cholest: Cholesterol
Comp: Components
CPK: Creatine phosphokinase
cps: capsule
creat: creatinine
slv: saliva
DHA: Docosahexaenoic acid
DNPH: 2,4-dinitrophenylhydrazine
DPPH: 1,1′-diphenyl-2-picrylhydrazyl
DRIv: Dietary Reference Intake values
EFSA: European Food Safety Authority
ELISA: Enzyme-linked immunosorbent assay
EE: Ethyl ester
En: Energy
EPA: Eicosapentaenoic acid
GPx: Glutathione peroxidase
HDL: High density lipoprotein
HGH: Human growth hormone
4-HNE: 4-hydroxy-2,3-trans-nonenal
HPA: Hypothalamo-pituitary-adrenal axis
HPT: Hypothalamo-pituitary-testicular axis
HRP: Horseradish peroxidase
IBD: Inflammatory bowel disease
IGF-1: Insulin-like growth factor
IL: Interleukin
LARN: *Livelli di Assunzione di Riferimento di Nutrienti ed energia per la popolazione italiana*
LDH: Lactate dehydrogenase
LPO: Lipoperoxidation
LMNEF: Low-molecular-weight non enzymatic fraction
MDA: Malondialdeyde
MHC: Major histocompatibility complex
MLSS: Maximal lactate steady state
N: Sample size
NDA: EFSA Panel on Dietetic Products, Nutrition and Allergies
8-OHdG: 8-hydroxy-2′-deoxyguanosine
OTS: Overtraining syndrome
PCA: Principal component analysis
PC: Principal components
PCc: Protein Carbonyl content
PPAR-γ: Peroxisome proliferator-activated receptor
PRI: Population reference intakes
PUFAs: Polyunsatured fatty acids
RBC: Red blood cells
RDA: Recommended Daily Allowance
RI: Reference Intake range for macronutrients
ROS: Reactive oxygen species
SD: Standard deviation
SED: Sedentary subjects
SINU: *Società Italiana di Nutrizione Umana*
SOD: Speroxide dismutase
S.r.l.: *Società a responsabilità limitata*
T: Testosterone
TNF-α: Tumor Necrosis Factor-α
trigly: triglyceride
urn: urine
WHO: World Health Organization
